# Dual Role of *DLK1* in GnRH Neuron Ontogeny

**DOI:** 10.1007/s12015-025-10972-y

**Published:** 2025-09-09

**Authors:** Nazli Eskici, Celia Gomez-Sanchez, Shrinidhi Madhusudan, Kristiina Pulli, Salla Keskitalo, Tanja Turunen, Kirsi Vaaralahti, Yafei Wang, Markku Varjosalo, Taneli Raivio

**Affiliations:** 1 Stem Cells and Metabolism Research Program (STEMM), Research Programs Unit, Faculty of Medicine, University of Helsinki, Helsinki, 00014 Finland; 2 Medicum, Faculty of Medicine, University of Helsinki, Helsinki, 00014 Finland; 3 Institute of Biotechnology, Helsinki Institute of Life Science HiLIFE, University of Helsinki, Helsinki, Finland; 4 Helsinki Proteomics Center, University of Helsinki, Helsinki, Finland; 5 Department of Biochemistry and Developmental Biology and Translational Cancer Medicine Program, Faculty of Medicine, University of Helsinki, Helsinki, Finland; 6 Pediatric Research Center, Helsinki University Hospital, New Children’s Hospital, Helsinki, 00014 Finland

**Keywords:** DLK1, GnRH neurons, CRISPR activation, Neural differentiation

## Abstract

Mutations in Delta Like Non-Canonical Notch Ligand 1 (*DLK1*), a paternally expressed imprinted gene, underlie central precocious puberty (CPP), yet the mechanism remains unclear. To test the hypothesis that *DLK1* plays a role in gonadotropin releasing hormone (GnRH) neuron ontogeny, 75 base pairs were deleted in both alleles of *DLK1* exon 3 with CRISPR-Cas9 in human pluripotent stem cells (hPSCs). This line, exhibiting More than 80% loss of DLK1 protein, was differentiated into GnRH neurons by dual SMAD inhibition (dSMADi), FGF8 treatment and Notch inhibition, as previously described, however, it did not exhibit accelerated *GNRH1* expression. Activation of (WT) *DLK1* with CRISPRa during dSMADi suppressed the formation of anterior neuronal precursors, and almost completely inhibited GnRH neuron fate. Instead, it induced the expression of Wnt pathway genes, markers of proliferating and non-proliferating neuronal progenitors, and dorsal spinal cord interneurons. Activation of *DLK1* expression, during the FGF8 phase, significantly increased *GNRH1* expression and secretion of GnRH decapeptide. In conclusion, significant loss of DLK1 protein did not accelerate *GNRH1* expression, suggesting that CPP caused by paternally inherited loss-of-function mutations is due to mechanisms acting up- or downstream of GnRH neurons. Conversely, timing of *DLK1* activation emerged as an important factor in determining GnRH neuron fate, with early expression disrupting GnRH lineage commitment and later activation enhancing *GNRH1* expression.

**Graphical Abstract:**

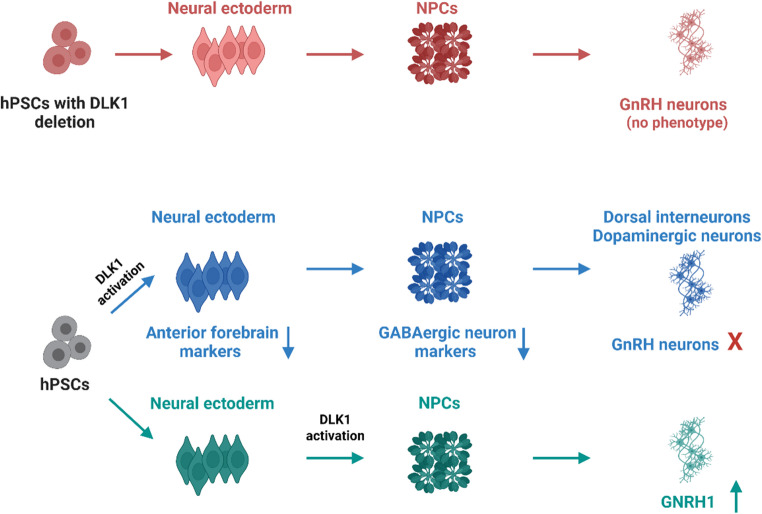

**Supplementary Information:**

The online version contains supplementary material available at 10.1007/s12015-025-10972-y.

## Introduction

*DLK1* is widely expressed during embryonic development and regulates cell proliferation and differentiation. Postnatally, its expression becomes restricted to the (neuro)endocrine tissues, stem/progenitor cells, and several hypothalamic nuclei, suggesting a role in the upstream regulation of GnRH production [1–3]. *DLK1* expression is highly dosage-sensitive in the brain, and thus alteration of expression levels can lead to impaired neural stem cell function and cognitive/psychiatric phenotypes [4]. It is also implicated in metabolic regulation, stem cell maintenance, and tumorigenesis [5].

*DLK1*, also known as fetal antigen 1 and preadipocyte factor 1, located on the human chromosome 14q32.2, is a maternally imprinted and paternally expressed gene [6, 7]. It belongs to the *DLK1-DIO3* imprinted gene cluster, which is associated with Temple syndrome (TS) and Kagami-Ogota syndrome [8–10]. The *DLK1-DIO3* locus contains the maternally expressed long non-coding RNAs (lncRNAs) *MEG3*, *MEG8*, and antisense *RTL1*, a large cluster of microRNAs (miRNAs), multiple small nucleolar RNAs (snoRNAs), and several pseudogenes as well as the paternally expressed protein-coding genes *DLK1*,* RTL1*, and *DIO3* [11]. Expression of this locus is tightly regulated by the intergenic differentially methylated region (IG-DMR) [1, 12]. Recently, Li et al. showed that the knockdown of *Meg3* decreased *GnRH1* and *Kiss1* expression in hypothalamic cells and delayed the puberty onset in rats [13].

DLK1 is a transmembrane and secreted protein. It contains six EGF-like motifs and lacks the Delta-Serrate-Lag1 motif (i.e., a non-canonical ligand) [14–16]. Therefore, DLK1 mainly inhibits Notch signaling in a competitive manner [17]; although there are also conflicting reports [18].

The onset of puberty triggers the activation of the hypothalamic-pituitary-gonadal (HPG) axis, resulting in the secretion of kisspeptin and gonadotropin-releasing hormone (GnRH). This hormonal cascade subsequently elevates the concentrations of luteinizing hormone (LH) and follicle-stimulating hormone (FSH), alongside sex steroids such as testosterone and estradiol [19, 20]. Before puberty onset, the HPG axis is transiently activated during embryonic development, after birth, and during the first few months of life. Following the quiescence of the HPG axis during childhood, the gradual reactivation of GnRH secretion in puberty brings about the acquisition of reproductive capacity and the attainment of sexual maturity [21]. The onset of puberty prior to the age of 8 years in girls or age of 9 years in boys is considered precocious and requires investigation to exclude underlying causes [22]. To date, there are only a handful of genetic causes known to underlie idiopathic central precocious puberty (CPP). Although CPP may be related to certain syndromes [23, 24], monogenic causes have been attributed to extremely rare gain-of-function mutations in *KISS1* and *KISS1R* [22, 25] that directly regulate GnRH neurons, as well as to loss-of-function mutations in *MKRN3* and *DLK1* [26, 27]. *MKRN3* inhibits the *GNRH1* expression and acts as a brake on the initiation of puberty. However, the role of *DLK1* has yet to be explained [28].

In this study, we examined the role of *DLK1* in the development of GnRH neurons from human pluripotent stem cells (hPSCs) by using the previously described differentiation protocol comprised of dual SMAD inhibition, administration of FGF8, and inhibition of Notch [29–31]. We probed the role of DLK1 ablation at the pace of GnRH neurogenesis. Subsequently, we generated a CRISPRa line that allows temporal upregulation of *DLK1* during the differentiation of human GnRH neurons from hPSCs.

## Materials and Methods

### Human Pluripotent Stem Cells

The H9 hESC line (WA09; WiCell) based H9C11 *GNRH1*-Tdtomato reporter cell line [30] was used to create a homozygous *DLK1* deletion hPSC line, and an inducible *DLK1* activation hPSC line. All the cell lines were maintained in Matrigel-coated dishes (Corning), using mTeSR1 culture medium (STEMCELL Technologies) at 37 °C and 5% CO_2_.

## Generation of DLK1 Deletion and Activation Lines

The *DLK1* deletion line was generated using the Gene Knockout Kit V2 (Synthego) according to the manufacturer’s instructions. Briefly, H9C11 *GNRH1*-TdTomato reporter cells (2 × 10^6^) [30] were electroporated with three single guide RNAs (sgRNAs) together with Cas9 nuclease, introducing a 75 bp deletion in *DLK1* exon 3 (*DLK1* c.183-257del) using the Neon transfection system (ThermoFisher Scientific, 1100 V, 20 ms, 2 pulses). The cells were then maintained in mTeSR1 containing 10µM ROCK Inhibitor (Selleckchem) and 10% CloneR (STEMCELL Technologies), and single-cell sorted 48 h after the electroporation using SONY SH800z sorter, and the colonies were screened for the deletion with PCR. Successful deletion and the loss of DLK1 protein were verified with Sanger sequencing and Western blot (WB).

The *DLK1* activation line was generated by electroporating the CRISPR activation hPSC line with sgRNAs targeting the *DLK1* 5’UTR. The sgRNAs were chosen from a previously published study [32]. CRISPR activation involves fusing the Cas9 protein without endonuclease activity (dCas9) with 12 VP16 activation domains and a dihydrofolate reductase (DHFR)-derived destabilization domain (DD), which uses trimethoprim (TMP) to stabilize the protein complex. The resulting dCas9VP192 complex can enhance transcription when co-expressed with guide RNAs targeting the gene of interest [33, 34]. To allow for the inducible activation of transcription, the dCas9VP192 complex was paired with a Tet-ON system [35]. In this way, the complex is stable only in the presence of doxycycline (dox) and trimethoprim (TMP). Thus, in the presence of both antibiotics and sgRNAs targeting the *DLK1* promoter region, this system allows temporal regulation of *DLK1* expression [34, 36]. Inducible *DLK1* overexpression was validated with qPCR and Western blot after 48 h of Dox and TMP treatment, respectively. The qPCR primers and antibodies are provided in Supplementary Tables 1 and 2.

## GnRH Neuron Differentiation

The *DLK1* deletion and the *DLK1* activation lines were differentiated into GnRH neurons using previously described GnRH differentiation protocol [29]. Briefly, at ~ 90% confluence of the stem cells, the protocol begins with dual SMAD inhibition [37] using N2B27 medium supplemented with 2 µM Dorsomorphin (Selleckchem) and 10 µM SB431542 (Sigma) for ten days, followed by ten days of 100 ng/ml FGF8 (Peprotech) treatment.

**Fig. 1:**
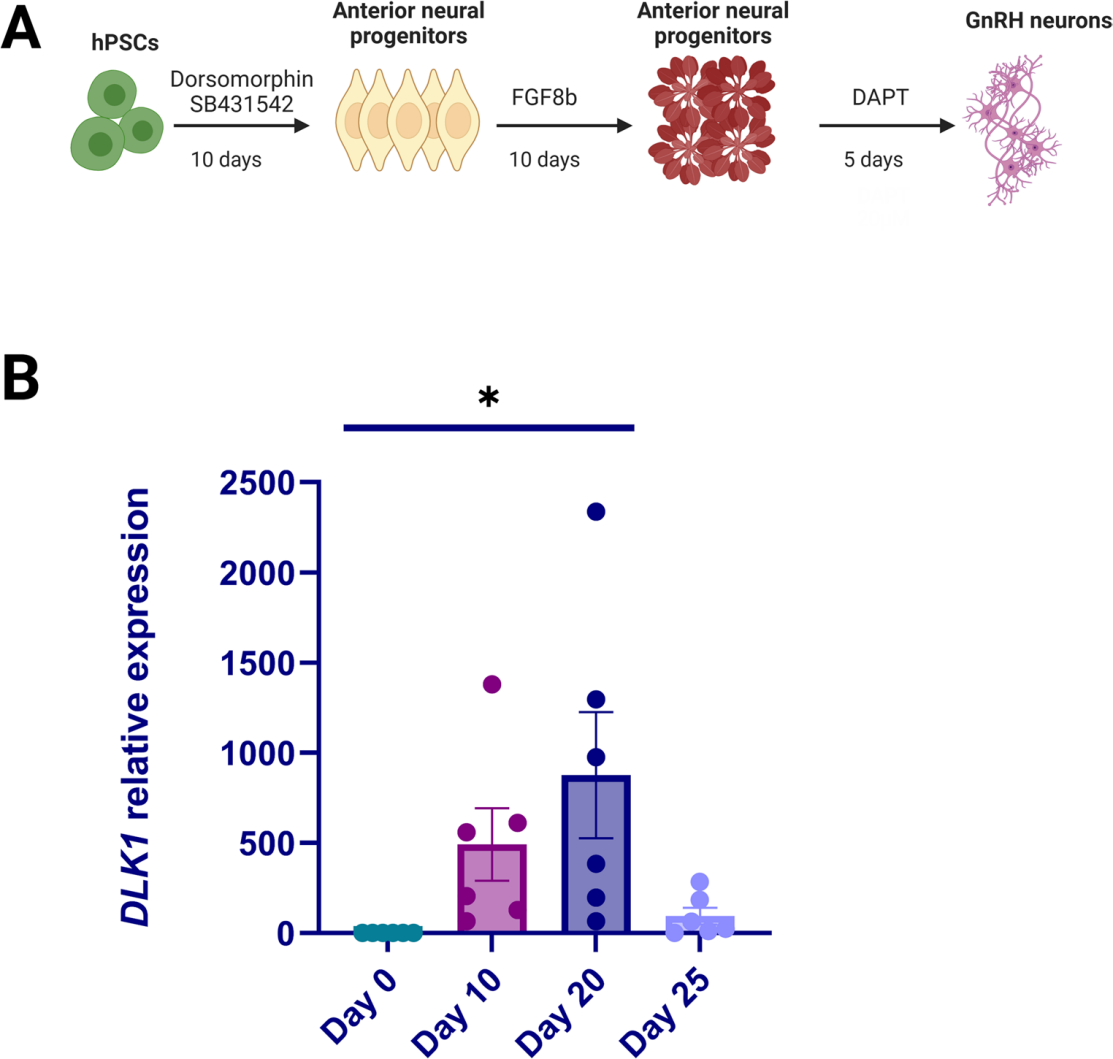
Endogenous *DLK1* expression during GnRH neuron differentiation. **(A)** Schematic of the GnRH neuron differentiation protocol consisting of 10-day dual SMAD inhibition, followed by ten days of FGF8 treatment, and subsequent Notch inhibition with DAPT for 5 days. **(B)** Endogenous *DLK1* expression on days 0, 10, 20, and 25 of GnRH neuron differentiation. *DLK1* expression increased from day 0 to day 20 (*n* = 6, * *p* <.05), meanwhile, the decrease from day 20 to day 25 approached statistical significance (*n* = 6, *p* =.06)

Subsequently, neurons are treated with 20 µM DAPT (Selleckchem) every other day for 5 days. The *DLK1* activation Line is further treated with 2 µg/ml Dox and 1 µM TMP to activate *DLK1* expression during the dSMADi phase or the FGF8 phase of the differentiation (Fig. 1A). Shortly, the cells were given Dox and TMP either together with dSMADi from day 0 to 10 (referred as condition acc), or together with FGF8 between days 10–20 (referred as condition cac), or throughout the differentiation from day 0 to day 25 (referred as condition aaa). Control cells with no Dox and TMP treatment are referred as condition ccc.

## Quantitative RT-PCR

RNA isolation was performed using Nucleospin RNA plus kit (Macherey-Nagel) and 1 µg of total RNA was reverse transcribed into cDNA using iScript cDNA Synthesis Kit (Bio-Rad). Quantitative PCR was performed using the primers Listed in Supplementary Table 1. Relative gene expressions were calculated using the 2^−ΔΔCt^ method by normalizing to Cyclophilin G (*PPIG*) expression.

## RNA Sequencing

RNA-sequencing was performed by Novogene GmBH, Munich, Germany. Samples were collected on days 10, 20 and 25. (See Supplementary Fig. 2 for details).

Shortly, messenger RNA was purified from total RNA using poly-T oligo-attached magnetic beads for the library preparation. After fragmentation, the first strand cDNA was synthesized using random hexamer primers, followed by the second strand cDNA synthesis using dTTP. The library was ready after end repair, A-tailing, adapter ligation, size selection, amplification, and purification [38]. The library was checked with Qubit and real-time PCR for quantification and bioanalyzer for size distribution detection. Quantified libraries were pooled and sequenced on Illumina PE150 platform, 20 million reads per sample, five repeats for each time point [38, 39].

### Western Blotting

The cells were lysed using RIPA buffer (ThermoFisher Scientific) supplemented with 1x protease and phosphatase inhibitors (ThermoFisher Scientific). Denatured proteins were loaded onto 4–12% SDS-PAGE gels (Bio-Rad), followed by transfer onto nitrocellulose membranes (ThermoFisher Scientific). The membranes were then blocked with 5% milk in 1x TBS-T at room temperature (RT), for 1 h. Primary antibodies were incubated overnight at 4 °C, while secondary antibody incubations were carried out at RT, for 1 h. Details of the primary and secondary antibodies are provided in Supplementary Table 2. Signals were detected using ECL Western Blotting Substrate (ThermoFisher Scientific) and visualized with the ChemiDoc Imaging System (Bio-Rad).

## ELISA

The culture media were collected from the *DLK1* activated cells (cac condition) on Day 25 to measure secreted GnRH decapeptide. LH-RH/Gn-RH Fluorescent EIA Kit (Phoenix Pharmaceuticals, FEK-040-02) were used according to the manufacturers’ instructions.

## Immunocytochemistry and Microscopy

Cells were fixed with 4% paraformaldehyde for 20 min at room temperature (RT). Following permeabilization with 0.5% Triton X-100 in 1x PBS for 10 min at RT, the cells were incubated with BlockAid Blocking Solution (ThermoFisher Scientific) for 1 h at RT. Primary antibodies were incubated overnight at 4 °C, while secondary antibody incubations were conducted in the dark at RT, for 1 h. Both primary and secondary antibodies were diluted in the blocking solution. Details of the antibodies used in this study are provided in Supplementary Table 2. Images were captured using a Zeiss Axio Imager.Z2 upright epifluorescence wide-field microscope.

### Sample Preparation for LC-MS/MS Analysis

Cell pellet was lysed using 200 µl of a solution consisting of 8 M urea (#424585000, Thermo Fisher Scientific) in 100 mM AMBIC (NH4HCO3, #A6141, Sigma Aldrich), sonicated for 20 min, and left to lyse on ice, with periodic vortexing for 2 h. Protein concentration was measured using Bio-Rad protein assay dye (#5000006, Bio-Rad Laboratories). Based on the results, 20 µg of protein was taken for in solution digestion. The samples were reduced with a final concentration of 5 mM Tris(2-carboxyethyl) phosphine hydrochloride (TCEP-HCl, #20490, Thermo Scientific) for 20 min, 37 °C with agitation. Alkylation was performed with iodoacetamide (#122271000, Acros Organics) in a final concentration of 10 mM and incubated for 20 min, at room temperature in the dark. The 8 M urea in the sample was diluted to 1 M using 100 mM AMBIC. The samples were digested O/N, in 37 °C with agitation using 1 µg of 1:1 Trypsin/Lys-C mix (V5073, Promega).

After digestion, the samples were desalted using Higgins Analytical columns (HUM S18V, Higgins Analytical) according to the manufacturer’s instructions.

### LC-MS/MS Analysis

The samples were resuspended in 60 µL of 0,1% formic acid (84865.26, VWR) buffer, diluted 1:10 and 4 µl of samples were loaded into Evotips according to the manufacturer’s instructions. For LC-MS/MS analysis, Orbitrap Astral MS (ThermoFisher Scientific) controlled with Thermo Tune software (version 1.1.477.46) coupled to an Evosep One (EvoSep Biosystems) was employed. The samples were analyzed with the 60 SPD method using an 8 cm x 150 μm Aurora Rapid analytical column (IonOptics) interfaced online using an EASY-Spray source. The column oven was set to 50 °C. Mobile phases A and B were 0.1% formic acid in water and 0.1% formic acid in acetonitrile.

The Orbitrap Astral MS was operated at a full MS resolution of 240,000 with a full scan range of 380–980 m/z. RF lens was set to 40%, and the full-MS AGC target was set to 300%. Maximum injection time for full-MS was 10 ms. MS/MS scans for DIA analysis were recorded with 3 Th isolation window from 380 to 980 m/z, 4 ms maximum ion injection time and AGC target 300%. The isolated ions were fragmented using HCD with 25% normalized collision energy.

Raw Orbitrap Astral DIA data were processed with DIA-NN version 2.2.0 [40]. A spectral library was generated from a human proteome FASTA file (downloaded on 14/05/2025 from UniProtKB/Swiss-Prot database, containing 20,383 proteins) using the following precursor ion generation parameters: Trypsin/P (protease), 1 missed cleavage, cysteine carbamidomethylation enabled as a fixed Modification, and N-terminal methionine excision was allowed as variable Modification, peptide length range from 7 to 30, precursor charge from 1 to 4, 380–980 m/z precursor range, and 120–1500 m/z fragment ion range. For the raw file searches, heuristic protein inference was enabled, and proteotypicity was set to protein names from FASTA. Mass accuracy was set to 10 and for 4 for MS1. Unrelated runs option was not selected, while match-between-runs were selected, and IDs, RT, and IM profiling was chosen for the library generation. Machine learning, quantification strategy, and cross-run normalization were set to cross-validated NNs, QuantUMS, and RT-dependent [41–43]. The false discovery rate for the output filtering was set to 1%. For data analysis the DIA-NN pr_matrix and pg_matrix were utilized [40, 44].

### Statistical Methods

For changes in *DLK1* expression from day 0 to day 20 (Fig. 1B), we used the summary measures method [45] and calculated Area Under the Curve (AUC) values for each differentiation from the corresponding time. The AUCs were compared to the AUC value of representing no change in expression over time with a two-tailed one-sample t-test. Relative expression of *DLK1* between day 20 and day 25 (Fig. 1B) was tested with a paired t-test. To investigate differences between conditions with different temporal activation of *DLK1*, we used one-way ANOVA with multiple comparisons. However, since the control condition was normalized to a constant value of 1.0 (removing its variance), comparisons against the control were carried out and reported using one-sample t-tests; otherwise, paired t-test was used. All the statistical tests were performed using GraphPad Prism (v9.2). Data are presented as mean ± SEM. A p-value less than 0.05 was accepted to indicate statistical significance.

For the bulk RNA sequencing, the raw data (raw reads) of fastq format were first processed through fastp software. All the downstream analyses were based on clean data with high quality. Index of the reference genome was built using Hisat2 v2.0.5 and paired-end clean reads were aligned to the reference genome using Hisat2 v2.0.5. The mapped reads of each sample were assembled by StringTie (v1.3.3b) in a reference-based approach. FeatureCounts v1.5.0-p3 was used to count the reads numbers mapped to each gene. Then FPKM of each gene was calculated based on the length of the gene and reads count mapped to this gene. Differential expression analysis of two conditions/groups (two biological replicates per condition) was performed using the DESeq2R package (1.20.0). The resulting P-values were adjusted using the Benjamini and Hochberg’s approach for controlling the false discovery rate. Genes with an adjusted P-value < = 0.05 found by DESeq2 were assigned as differentially expressed [46–49]. To improve the reliability of differentially expressed gene (DEG) detection, we applied a filtering step to remove genes with below 100 read counts. This was done to prevent spurious fold changes arising from lowly expressed genes with minimal biological significance. As a result, the final DEG list includes genes with sufficient expression levels for robust statistical analysis.

## RESULTS

### DLK1 Protein Is Efficiently Depleted in the DLK1 Deletion Line

**Fig. 2:**
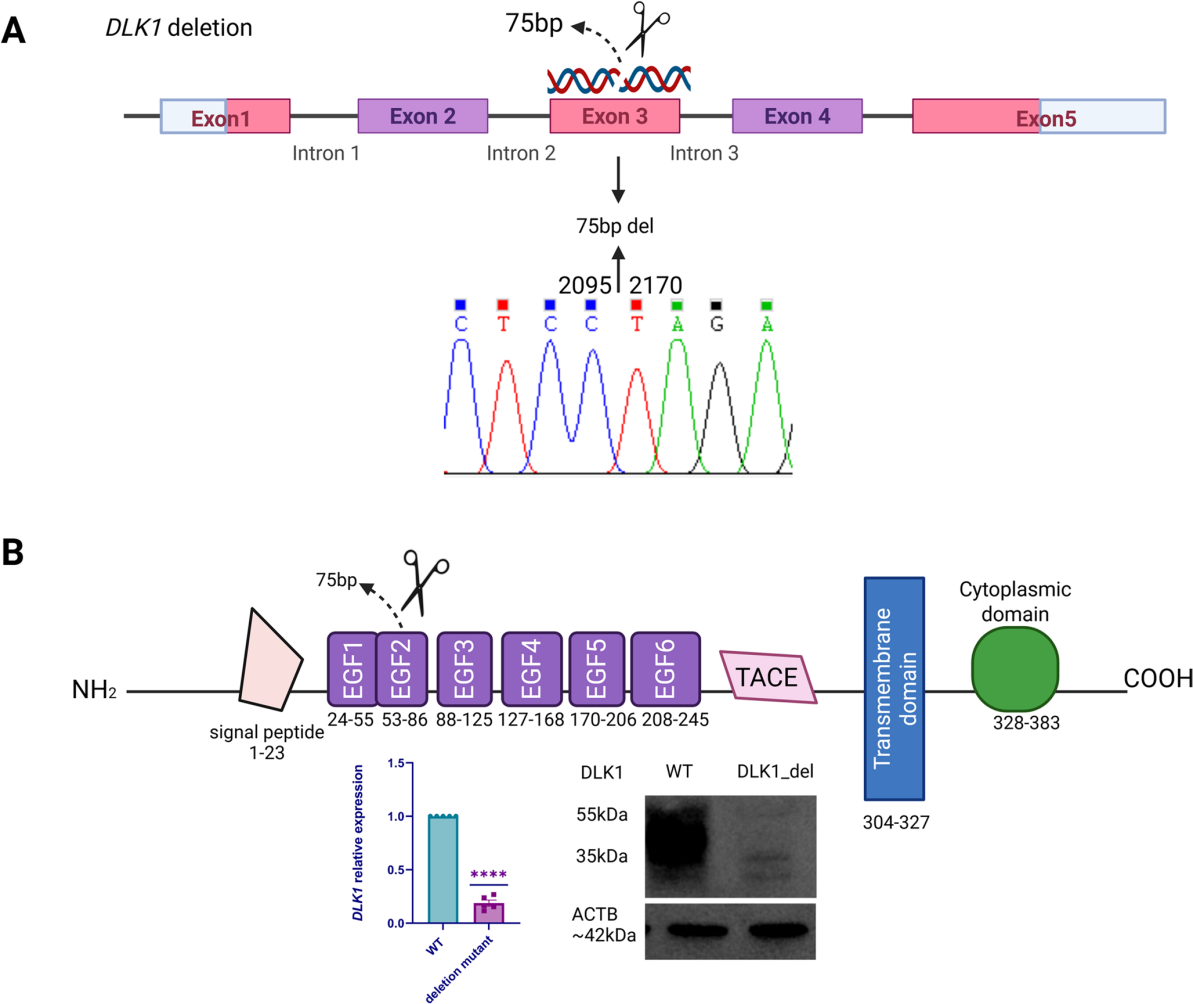
Generation and validation of *DLK1* deletion line. **(A)** 75 bp deletion was created in H9 GnRH-TdTomato reporter hPSCs by electroporating three sgRNAs targeting exon 3 of human *DLK1*, together with Cas9 nuclease. The presence of a homozygous deletion was confirmed by Sanger sequencing. **(B)** Schematic displays the DLK1 carrying a homozygous 75 bp deletion in exon 3. *DLK1* qPCR and Western blot analysis show the loss of DLK1 mRNA (*n* = 5, **** *p* <.0001) and protein (~ 45 kDa), respectively. ACTB (~ 42 kDa) was used as a loading control in WB

To assess the role of DLK1 loss in GnRH-neuron development, we generated a deletion of 75 bp in *DLK1* exon 3 by CRISPR/Cas9 (Fig. 2A). We used the H9C11 *GNRH1*-Tdtomato reporter hESC line [30] that allows identification of *GNRH1*-expressing cells in the cultures. The homozygous deletion in the *DLK1* deletion line was verified by Sanger sequencing (Fig. 2A). *DLK1* qPCR, and WB of DLK1 showed a drastic reduction of *DLK1* mRNA and DLK1 protein compared to WT cells, respectively (Fig. 2B, Supplementary Fig. 5).

We next verified the loss of protein expression with LC-MS/MS analysis. Data-independent acquisition (DIA) profiling on the Orbitrap Astral platform robustly quantified the proteomes of WT and *DLK1* deletion lines [40]. Across the dataset, the most striking genotype-dependent difference was observed for DLK1 (UniProt P80370). In WT cells, the summed precursor-level intensity for DLK1 reached 2.10 × 10^7 arbitrary units, whereas, in the deletion Line, the corresponding intensity dropped to 4.00 × 10^6 (data not shown).

This represents an ≈ 5.3-fold decrease (≈ 81% reduction) in DLK1 signal in the deletion Line relative to WT, confirming that the CRISPR-mediated deletion of exon 3 almost completely abolishes DLK1 protein expression. The residual low-level signal detected in the deletion line samples is > 10-fold lower than WT.

No widespread proteome remodeling was evident beyond DLK1 itself, indicating that the deletion procedure selectively impacts DLK1 while leaving the broader protein landscape largely intact.

### Loss of DLK1 Protein Does not Accelerate GnRH Neuron Differentiation

**Fig. 3:**
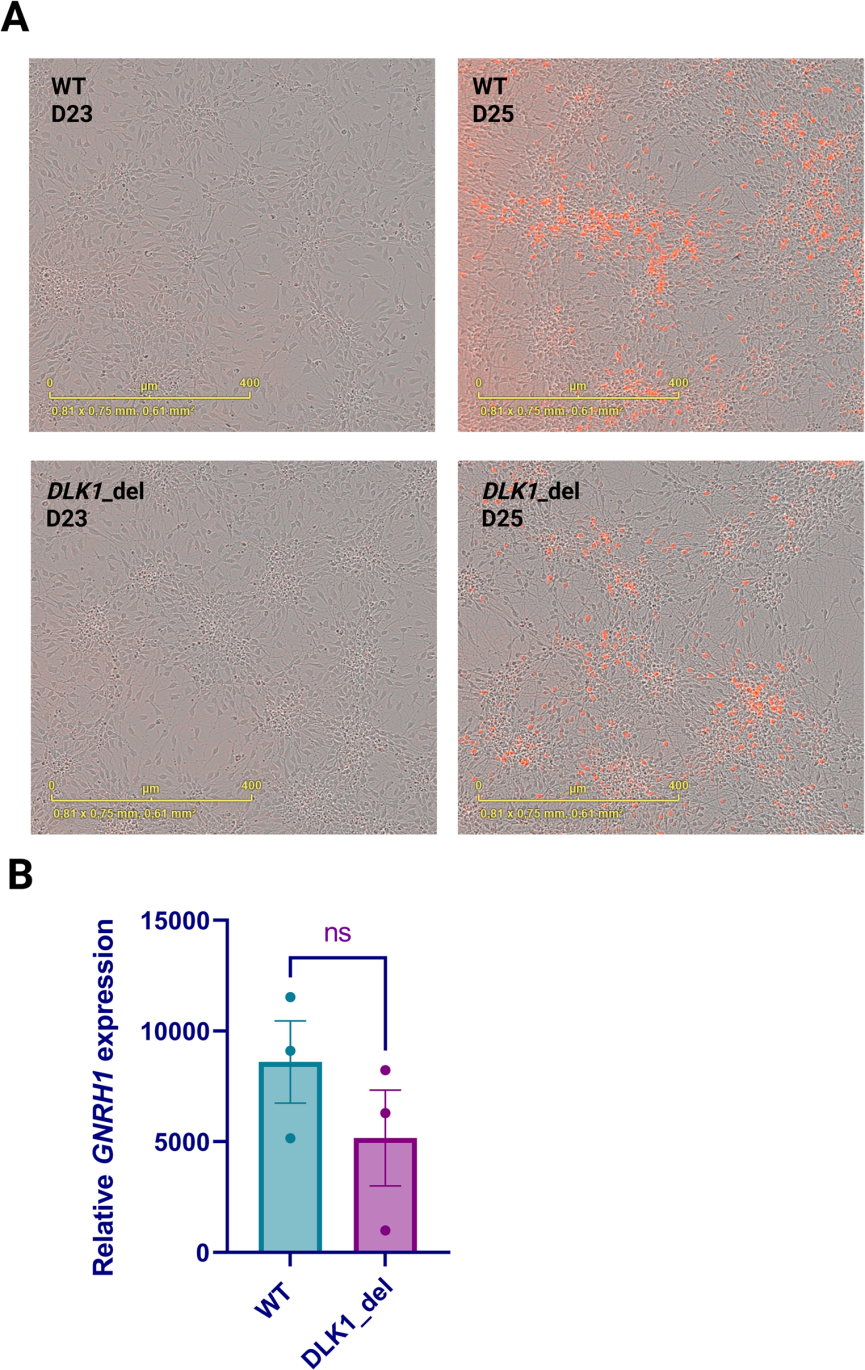
*DLK1* deletion line can differentiate into GnRH-expressing neurons. **(A)** Day 23 and day 25 images of WT and *DLK1* deletion GnRH-TdTomato reporter line at the GnRH stage of the differentiation. The red cells represent *GNRH1* expression. *GNRH1*-expressing TdTomato-positive neurons emerged no earlier than D25 in both WT and *DLK1* deletion line. **(B)** Relative *GNRH1* expression on Day 25 was comparable between WT and *DLK1* deletion line (*n* = 3). The expression levels are calculated compared to day 0

We differentiated human pluripotent stem cells into GnRH neurons using the previously established protocol [29] (Fig. 1A). During the differentiation, *DLK1* was highly expressed in neural progenitors at both day 10 (D10) and day 20 (D20). However, its expression declined nearly significantly (*p* =.06) in day 25 (D25) terminally differentiated neurons, though some expression persisted (Fig. 1B). At the end of the differentiation, both the *DLK1* deletion line and WT cells were able to produce TdTomato-expressing GnRH neurons. GnRH neuron formation was not accelerated in the *DLK1* deletion Line as there were no TdTomato-positive cells were observed on day 23 (Fig. 3A). Additionally, there were no significant differences in *GNRH1* expression between the *DLK1* deletion cells and the WT cells on day 25 (Fig. 3B). Thus, loss of More than 80% of DLK1 protein due to the homozygous deletion in *DLK1* exon 3 does not accelerate GnRH neuron formation and/or its expression in our in vitro differentiation model.

### DLK1 Has a Time-Dependent Effect on Neural Fate Determination

**Fig. 4:**
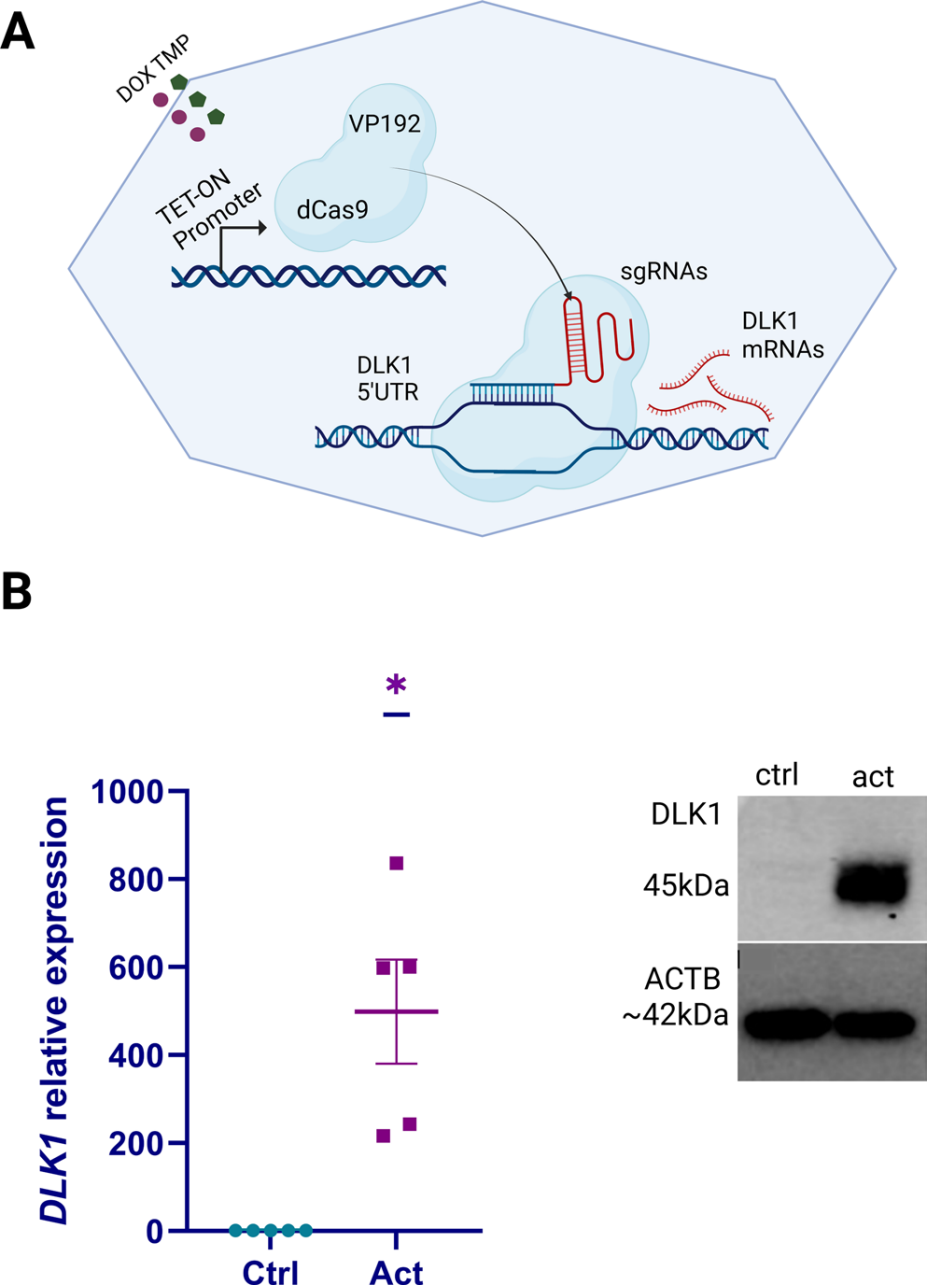
Generation and validation of *DLK1* activation line. **(A)** Inducible dCas9VP192 complex along with two sgRNA guides targeting the *DLK1* 5’UTR were electroporated into *GNRH1*-TdTomato reporter hPSC [29]. Addition of Doxycycline (Dox) and trimethoprim (TMP) activates and stabilizes the complex, respectively, which in turn induces *DLK1* expression. **(B)** qPCR and Western blot show increased expression of DLK1 mRNA (left, *n* = 5, * *p* <.05) and protein (right) after 48 h of simultaneous Dox and TMP treatment, respectively. The expression levels are calculated compared to non-activated cells

**Fig. 5:**
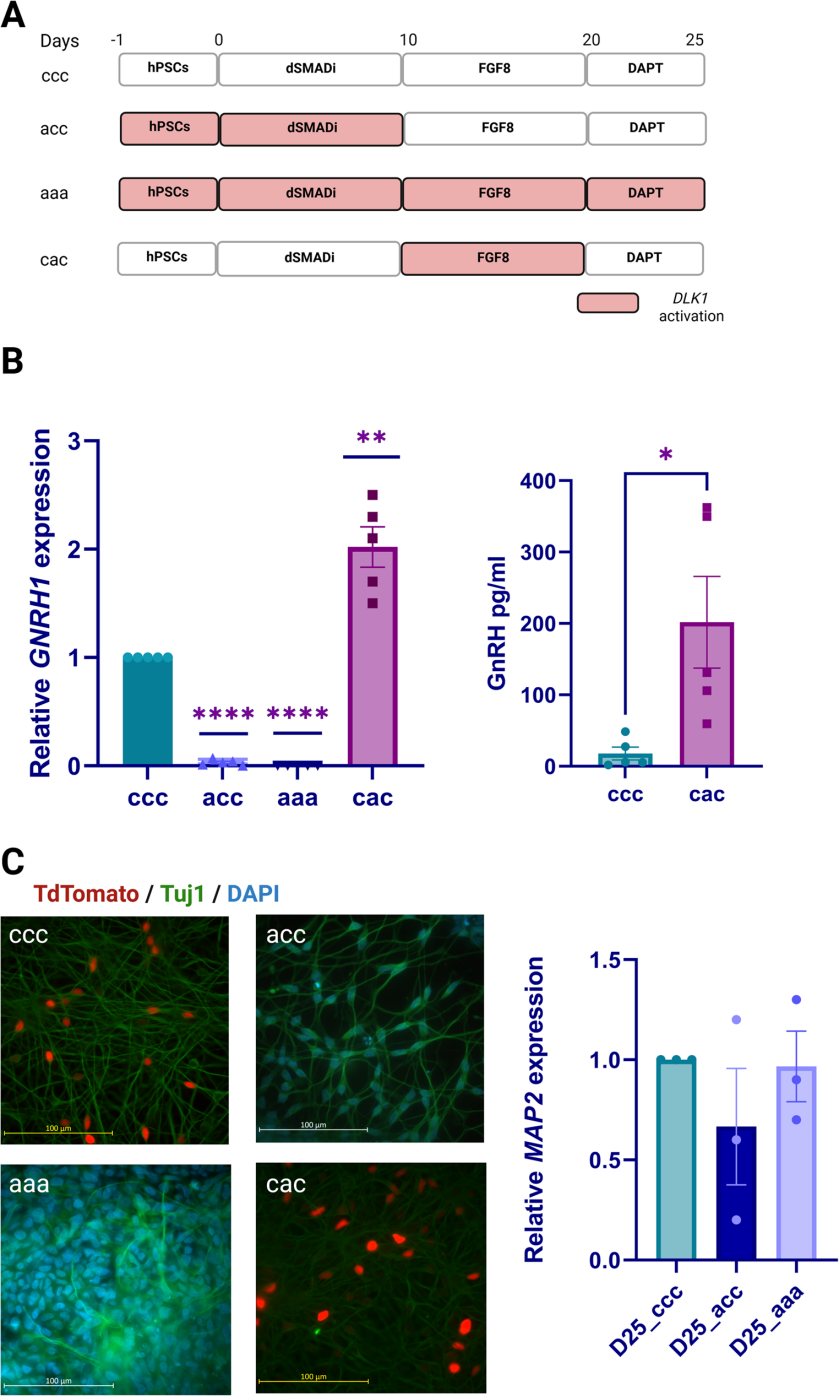
Differentiation of *DLK1*-activated cells into GnRH neurons. **(A)** Schematic of the *DLK1 *activation during GnRH neuron differentiation. Conditions: No activation (ccc), *DLK1* activation during dSMADi (acc), *DLK1* activation throughout the protocol (aaa), *DLK1* activation during FGF8 treatment (cac). **(B)** *GNRH1* expression (left) and GnRH decapeptide secretion (right) were assessed by qPCR and ELISA, respectively. *DLK1* activation during dSMADi between the days 0–10 (acc), or throughout the protocol days 0–25 (aaa) abolished *GNRH1* expression, while activation during FGF8 phase between days 10–20 (cac) increased *GNRH1* expression and GnRH secretion compared to the non-activated control cells (ccc) (*n* = 5, * *p* <.05, ** *p* <.01, **** *p* <.0001). **(C)** GnRH neuron numbers (TdTomato positive cells) were significantly decreased in 10-day (acc) or 25-day activated (aaa) conditions on day 25, unlike in the no-activation (ccc) or activation during the FGF8 phase (cac) conditions. TUJ1 immunostaining (left) and *MAP2* qPCR (right) at day 25 of differentiation were used as a marker for neuronal differentiation in all conditions (Green: TUJ1, Red: GnRH-TdTomato, Blue: DAPI)

Next, we generated a *DLK1* activation hESC line (Fig. 4A), using H9C11*GNRH1*-Tdtomato reporter cells, and validated the increased *DLK1* mRNA and protein levels at the stem cells stage with qPCR and WB, respectively (Fig. 4B). We then activated *DLK1* expression in different stages of the GnRH-neuron differentiation protocol (Fig. 5A). When *DLK1* was activated during the dSMADi phase (days 0–10; acc) or throughout the protocol (days 0–25; aaa), there was a drastic reduction of *GNRH1* expression at the end of the protocol when compared to the no-activation control (ccc) (Fig. 5B). Conversely, activation of *DLK1* only during the FGF8 phase (cac) increased expression of *GNRH1* on day 25 (Fig. 5B). Consistent with the observed expression pattern of *GnRH1*, there was a significant increase of GnRH in the culture media on day 25 cac condition (Fig. 5B). In contrast, GnRH levels in both acc and aaa condition media were found to be below the detection level (data not shown). We did not observe any TdTomato positive cells in 10-day (acc) or 25-day activated (aaa) conditions on day 25 (Fig. 5C). Instead, the no-activation (ccc) and activation during the FGF8 phase (cac) conditions both produced TdTomato positive cells (Fig. 5C). The overall neurogenesis was not affected in acc and aaa conditions, since all the conditions expressed neuronal markers TUJ1 and *MAP2* (Fig. 5C). Given that *DLK1* is part of an imprinted locus whose expression is tightly regulated, we wanted to assess its expression pattern during dSMADi under activation conditions [4, 50–52]. Interestingly, we observed that the levels of *DLK1* expression gradually diminished during dSMADi (Supplementary Fig. 1A) despite the sustained high levels of dCas9 expression due to the continuous treatment with doxycycline (Dox) and trimethoprim (TMP) (data not shown). These results suggest that continuous *DLK1* activation led to dynamic temporal changes in *DLK1* expression levels during the dSMADi phase, with initial overexpression occurring during the first days of activation, followed by a downregulation around day 5 that persisted until day 20. Although some expression remained, it was lower than that observed in the non-activated control cells on both day 10 and day 20 (Supplementary Fig. 1A-B).

Instead, when *DLK1* was activated after dSMADi during the FGF8 treatment, the effect of activation did not diminish, and *DLK1* was still upregulated on D20 of the cac condition (Supplementary Fig. 1B).

### DLK1 Overexpression Alters the Transcriptomic Profile of the Neural Progenitor Pool and Neuron Populations

**Fig. 6:**
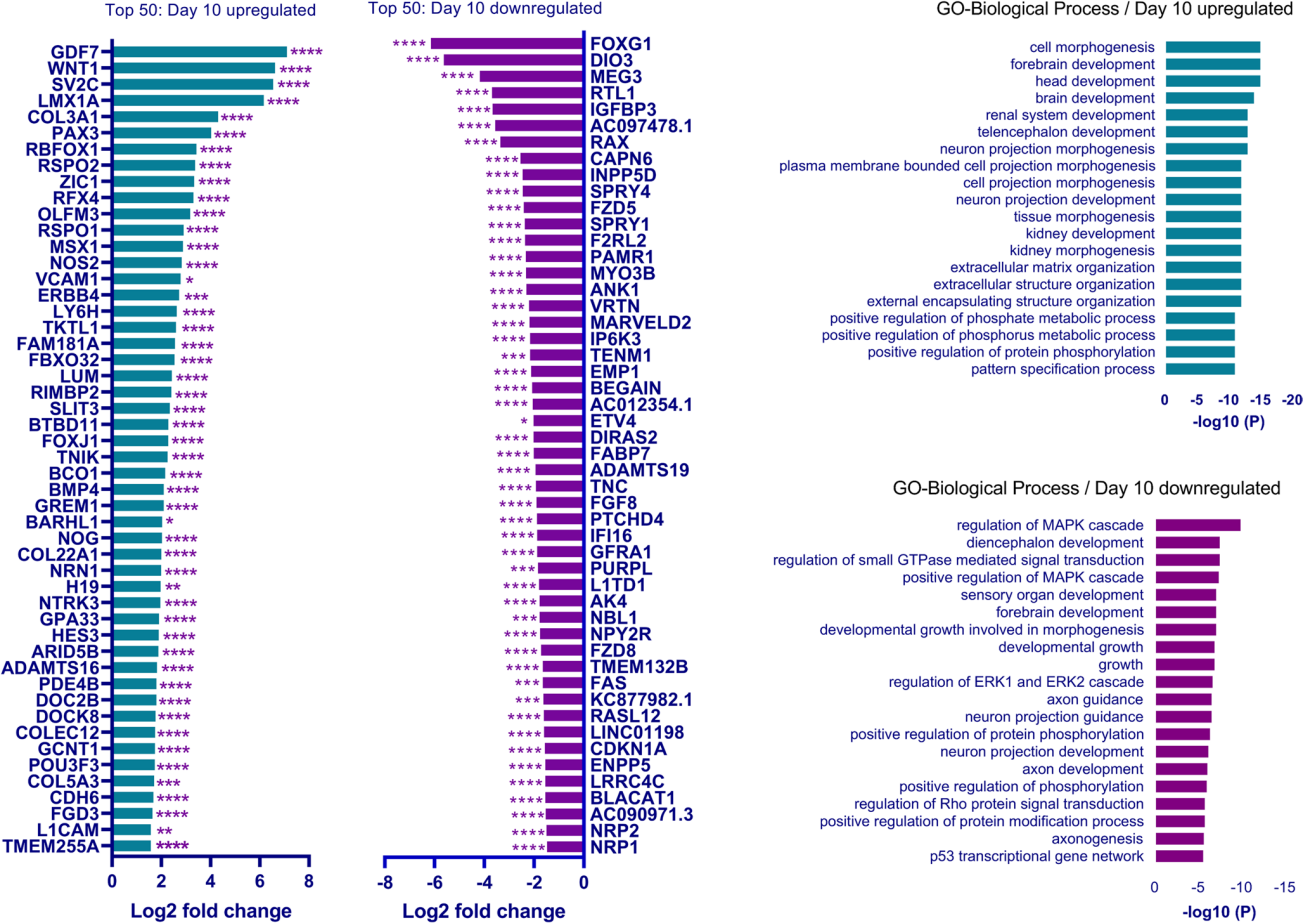
Top 50 differentially expressed genes (DEGs) on day 10 when *DLK1* was activated during dSMADi. Left panel shows the top 50 upregulated and downregulated genes, right panel shows the top 20 GO terms of the D10 DEGs (* *P* <.05, **P <.01, ***P <.001, **** *P* <.0001)

To elucidate the effects of *DLK1* overexpression on neuronal fate, we assessed the transcriptomic changes in activated cells on differentiation days 10, 20 and 25 by RNA sequencing (Supplementary Fig. 2). The first step of the GnRH neuron differentiation protocol (dSMADi) produces anteriorly patterned cells that express neural progenitor markers such as *FOXG1*, *SOX2*, and *PAX6* by blocking BMP and TGF-β/activin signaling pathways [29, 37]. After filtering out the differentially expressed genes with read counts below 100, there were 696 genes differentially expressed on day 10, out of which 412 were upregulated and 284 were downregulated (Supplementary Table 3). While this filtering step reduced the total number of differentially expressed genes (DEGs), it minimized the inclusion of low-confidence genes with very low expression levels, leading to a more robust interpretation of *DLK1*-dependent transcriptional changes. The anterior neural progenitor markers *FOXG1*, *SOX2*, and *FGF8* were significantly downregulated, while Wnt pathway genes *WNT1*,* RSPO1*,* RSPO2*, and *TNIK* were significantly upregulated (Table 1). The top 50 DEGs and Top 20 GO terms are shown in Fig. 6. Interestingly, in spite of continuous Dox and TMP treatment, the *DLK1-DIO3* locus genes (*DIO3*,* RTL1*,* MEG3*) were in the top 50 downregulated gene list (Fig. 6).

**Table 1:** Overview of the differentially expressed genes on days 10, 20, and 25 with 10-day (dSMADi, acc) or 25-day (whole protocol, aaa) *DLK1* activation. In both acc and aaa conditions, anterior forebrain markers on D10 and GABAergic neuron progenitor markers on D20, which indicate GnRH neuron fate, were downregulated. In line with this, GABAergic neuron and glutamatergic neuron genes were downregulated on D25. In contrast, Wnt and Notch genes were upregulated at all time points and on D25, respectively, in both acc and aaa conditions

	Day 10	Day 20	Day 25
**↓**Anterior forebrain markers (dSMADi genes)	FOXG1, LHX2, FGF8, DMRTA1, RAX, FABP7, PURPL, FZD5, CAPN6, GFRA1, SOX2		
**↓**GABAergic neuron progenitor markers		GAD1, GABRA5, SLC32A1, SCGN, OLIG2, DLX1/2, GSX2, VAX1	
**↓** GABAergic neuron markers (GnRHGABA cluster) (31)			GNRH1, ISL1, DSEL, C11orf96, DLX2, VGF, DLX6, ECEL1, SLX5, GAD2, DLX1, SLC32A1, SPOCK1, GRIA2, NRXN3, ALCAM, PPP2R2B, RASD1, FGF14, UNC5D, FGF3, FGF19, GRID2, SCGN
**↓** Glutamatergic neuron markers (31)			SCN2A, RELN, AR, GABRA2, PPP1R17, PPP2R2B, CDH12, PPP2R2C
**↑** Neural crest/dorsal neuron/sensory neuron markers	GDF7, BMP4, GREM1, NOG, PAX3, MSX1, ZIC1	LMX1A, BMP6, GDF7, NEUROG2	LMX1A, DMRT3, BHLHE22, OTX1, NEUROG2, SPARC
**↑** WNT pathway genes	WNT1, RSPO1, RSPO2, TNIK	WNT5A, WNT7B, WNT8B, WLS, RSPO2, RSPO3, BAMBI	WNT5A, WNT7B, WNT8B, WNT10B, WLS, RSPO2, LEF1, FZD7, LGR5, BAMBI
**↑** Notch pathway genes			NOTCH2, DLL4, NOTCH3, NOTCH1, NRARP, DLL3, DLL1, JAG1, HES1, HES6, HEY1, LFNG, MFNG, SOX2, PAX6, NR2E1, NEUROG1, NEUROG2

During the second (FGF8) phase of the GnRH differentiation, the cells proliferate and become primed to make anterior forebrain neurons. On day 20, *DLX1/2* and *DLX5/6* transcription factors are highly expressed together with GABAergic neuron progenitor markers [29]. When *DLK1* was activated during dSMADi (acc condition), there were 2217 DEGs on day 20 out of which 1228 were upregulated and 989 were downregulated (Supplementary Table 4). The DEG list for *DLK1* activation throughout the whole protocol (aaa condition) was almost the same as acc list. Several Wnt pathway genes, including *WNT5A*,* WNT7B*,* WNT8B*,* WLS*,* RSPO2*,* RSPO3*, and *BAMBI*, were upregulated in acc and aaa conditions. Conversely, GABAergic progenitor markers including *GAD1*,* GABRA5*,* SLC32A1*,* SCGN*,* OLIG2*,* GSX2*,* VAX*, and the *DLX* family genes *DLX1/2* and *DLX5/6* were significantly downregulated along with the *DLK1-DIO3* locus genes (Table 1).

Finally, at the third stage of the protocol, inhibiting Notch signaling with DAPT produces two main neuronal populations: GABAergic GnRH neurons that express *ISL1* and the *DLX* family genes, and excitatory glutamatergic neurons that express protein phosphatase regulatory subunit family genes (*PPP1R17*,* PPP2R2B*, and* PPP2R2C*) [30]. The transcriptomic profile of the day 25 cells was drastically changed when we activated *DLK1* from the stem cell stage until the end of dSMADi (until day 10, condition acc) or throughout the protocol (from day 0 to 25, condition aaa), as revealed by principal component analysis (Supplementary Fig. 3). With *DLK1* activation during dSMADi (acc), 2610 genes were differentially expressed on day 25 out of which 1501 were upregulated and 1109 were downregulated (Supplementary Table 5). Both glutamatergic and GABAergic neuron markers were significantly downregulated in acc and aaa conditions. Instead, these conditions gave rise to TUJ1-positive neurons (Fig. 5C) that express dorsal spinal cord interneuron markers *LMX1A*,* BHLHE22*, and *DMRT3* [53–55], and the neural crest cell markers *SPARC*,* TFAP2A*,* ID1*, and *ID2.* Additionally, several of the Wnt and Notch pathway genes were also upregulated. Notably, *GNRH1* was the Most downregulated gene on day 25 in acc and aaa conditions (Table 1). qPCR validation of differentially expressed genes is shown in Supplementary Fig. 4.

Conversely, when we activated *DLK1* during the FGF8 stage (from day 10 to 20, condition cac) the List of differentially expressed genes on day 25 was very small, 9 genes upregulated, and 31 genes downregulated. The genes relevant for hypothalamic differentiation such as *NHLH2* and *TENM1* were slightly upregulated in the cac condition.

## Discussion

Two of the four so far described genes linked to monogenic CPP are imprinted genes, suggesting that imprinting may have a role in the regulation of puberty [56, 57]. Imprinted genes play a significant role in early development by modulating growth pathways and are vital for the balanced maturation of organs [58]. *DLK1*, a paternally expressed imprinted gene, is an integral component of the imprinted gene network [5, 14]. It belongs to the *DLK1-DIO3* imprinted gene cluster, which consists of three paternally expressed protein-coding genes: *DLK1*,* RTL1*, and *DIO3*, and several maternally expressed non-coding transcripts, such as *MEG3* and *MEG8.* This gene cluster plays a crucial role in the normal development of both humans and mice [59]. Karami et al. reported that in bovine models, *DLK1-DIO3* locus genes were differentially expressed in the hypothalamus and pituitary gland of pre- and post-pubertal animals [60]. Furthermore, Mo et al. showed that loss of expression of the *DLK1-DIO3* lncRNAs caused reduced neural differentiation from hESCs [61], and Li et al. showed that knockdown of *Meg3* in female rats results in downregulation of *GnRH* and *Kiss1* expression strengthening the notion of this imprinted locus may regulate the onset of puberty [13].

To date, all reported mutations in *DLK1* associated with CPP have been characterized as loss-of-function (LOF) mutations, which result in the production of truncated or non-functional proteins, indicating that *DLK1* acts as a puberty brake [3]. The evidence so far shows that DLK1 is most abundant in early embryogenesis, overlapping temporally with the origin and migration of GnRH neurons [16, 62]. *Dlk1* is shown to be expressed in the hypothalamus and is proposed to have a role in the postnatal maturation of hypothalamic neurons [16]. As embryonic development proceeds, *DLK1* expression decreases, while GnRH neurons complete their migration and begin functional maturation. Therefore, the decrease in *DLK1* levels leading to Notch activation may help GnRH neurons exit the progenitor pool and migrate into the brain. However, direct experimental data linking *DLK1* timing to GnRH neurogenesis are scarce. While previous studies have shown that *DLK1* loss-of-function is the cause of precocious puberty in patients with Temple syndrome [17, 63, 64] and proposed roles in neuroendocrine regulation, our study is the first to functionally dissect *DLK1*’s role in human GnRH neuron development using an hPSC-based model. The *DLK1* deletion Line, carrying a 75 bp in-frame deletion in *DLK1*, showed markedly reduced *DLK1* mRNA levels and loss of DLK1 protein, as evidenced by Western blot using two different antibodies targeting the C-terminal region of the protein, and an ~ 81% reduction in DLK1 protein levels by LC-MS. Thus, this line was suitable for investigating the effect of significant DLK1 depletion on GnRH neuron ontogeny. Expectedly, DLK1 was dispensable for GnRH neuron differentiation in vitro. Surprisingly, however, DLK1 depletion did not accelerate their maturation. This suggests that LOF mutations in *DLK1* cause CPP by regulating the signaling pathways associated with the function of GnRH neurons and/or GnRH signaling at the pituitary level [65].

We next investigated an opposite effect, i.e., that of a temporal activation of *DLK1* on GnRH neuron ontogeny. During the initial phase of our GnRH neuron differentiation protocol, hPSCs are treated with dSMADi for 10 days to induce anterior neural fates characterized by the expression of *FOXG1*,* SOX2*, and *LHX2* [29, 31]. Activation of *DLK1* during dSMADi (acc) markedly reduced the expression of these and other anterior forebrain progenitor markers (see Table 1) and ultimately compromised the formation of GnRH neurons.

In day 25 cells of 10-day activated (acc), and 25-day activated (aaa) conditions, glutamatergic (*PPP1R17*,* TBR1*,* LHX1*,* LHX2*,* LHX5*,* LHX9)* and GABAergic (*GNRH1*,* GAD2*, *DLX1*,* DLX2*,* DLX5)* neuron markers were downregulated, whereas proliferating (*HMGB2*,* MKI67*,* TOP2A)* and non-proliferating (*NEUROG1*,* NEUROD1/4*,* SOX2)* progenitor markers were upregulated [31]. This could result from a delayed differentiation and a shift toward neural progenitor states. This also supports a role for *DLK1* in sustaining a slow-dividing stem cell pool and delaying neurogenesis, as previously reported [4]. Additionally, several studies have shown the inhibitory effect of *Dlk1* overexpression on differentiation in various tissues [2, 16, 63, 66, 67]. Falix et al. showed that constitutive overexpression of soluble DLK1 prevents adipogenic differentiation in mice by inhibiting the expression of the key transcriptional regulators of adipogenesis [68]. Mirshekar-Syahkal reported that overexpression of *Dlk1* does not appear to negatively influence cell survival or hematopoietic stem cell (HSC) generation, but it affects their function and maintenance, as evidenced by reduced stem cell activity despite increased proliferation in *Dlk1*-transgenic embryos [69]. However, the effect of *Dlk1* overexpression on brain tissue has yet to be investigated. Additionally, several sensory interneuron markers such as *LMX1A*,* DMRT3*, and *BHLHE22* were upregulated in day 25 acc and aaa cells, suggesting a shift towards a more dorsal phenotype [70]. The alterations in *DLK1* levels may lead to hyperactivation of Notch signaling, and consequent inhibition of ventral cell differentiation and changes in cell fate determination [71–73], as Notch signaling is critical for maintaining the balance between dorsal and ventral cell fates [74]. Indeed, we observed upregulation of several Notch genes in day 25 cells of acc and aaa conditions (Table 1). Furthermore, upregulation of Wnt signaling during dSMADi inhibition in acc and aaa conditions may have inhibited the ventralization and promoted expression of dorsal telencephalic markers [75].

Of note, *GNRH1* was the Most downregulated gene on day 25 in both conditions where *DLK1* was activated during dSMADi (acc, aaa), suggesting that early *DLK1* activation has a strong negative effect on GnRH fate. Along with *GNRH1*, several genes implicated in GnRH neuron ontogeny such as *SIX3*, *DLX1/2*,* DLX5/*6, and *ISL1* [30, 76–78] were also significantly downregulated at the end of the differentiation.

It is noteworthy that in the *DLK1-DIO3* cluster, the protein-coding genes *DLK1*, *DIO3* and *RTL1*, as well as the long non-coding RNAs *MEG3* and *MEG8*, were significantly downregulated when *DLK1* was activated during dSMADi phase (acc, aaa). This observation suggests that the *DLK1-DIO3* locus genes are regulated together and, in our study, attempts to overexpress *DLK1* resulted in the downregulation of the entire *DLK1-DIO3* cluster (Supplementary Fig. 4). While *MEG3* downregulation in our *DLK1*-activated cells may contribute to impaired GnRH neuron development, current evidence for the effect of *MEG3* on GnRH neuron ontogeny/function remains indirect. *Meg3* has been shown to regulate *Kiss1* expression via epigenetic mechanisms and to influence GnRH neuron function and secretion [13, 79]. Moreover, epigenetic changes in the *MEG3* locus have been associated with CPP [80–82]. However, a direct mechanistic role for *MEG3* in GnRH neuron lineage specification remains to be established.

The drastic effects of *DLK1* activation in the early stage of the differentiation on GnRH neuron ontogeny prompted us to investigate the effects of *DLK1* activation following the dSMADi phase. When *DLK1* was activated exclusively in the FGF8 phase (cac), *GNRH1* expression and GnRH decapeptide in culture media both increased at d25 (Fig. 5B). Of note, *DLK1* activation during dSMADi (acc) or dSMADi and FGF8 phase (aaa) both resulted in the absence of GnRH neurons (Fig. 5C). Early *DLK1* activation coincides with dual-SMAD inhibition and disrupts anterior neural progenitor fate, potentially via Wnt pathway activation and downregulation of *FOXG1* and *DLX* genes [31]. In contrast, *DLK1* activation during the FGF8 phase appears to enhance GnRH neuron development, possibly by promoting responsiveness to anterior patterning signals. While it remains unclear whether *DLK1* acts upstream or downstream of these signaling pathways, our transcriptomic data suggest that *DLK1* may act as a context-dependent modulator rather than a direct effector of a single pathway. Additionally, the neural fate determination seems to occur in the very early days of differentiation, and *DLK1* levels may influence whether cells become dorsal or ventral neurons.

In conclusion, our study is the first to dissect the role of *DLK1* in human GnRH neuron development using an hPSC-based model. Our *DLK1* deletion line, which mimics the loss-of-function mutations seen in CPP patients with More than 80% loss of DLK1 protein, did not show accelerated GnRH neuron differentiation. This suggests that CPP associated with *DLK1* mutations may arise from mechanisms related to pituitary and/or hypothalamic regulation of GnRH signaling. However, increased doses of *DLK1* at different stages of our protocol significantly altered GnRH neuron fate, with its early activation disrupting GnRH neuron formation, whereas overexpression during the FGF8 phase enhancing *GNRH1* expression and GnRH decapeptide production. These findings point to the importance of further investigating the role of *DLK1-DIO3* locus genes in GnRH neuron development and the interaction between *DLK1* and other regulators of puberty, such as pituitary gonadotropes, and hypothalamic kisspeptin neurons.

## Supplementary Information

Below is the link to the electronic supplementary material.

Supplementary Figure 1 *DLK1* expression during dSMADi and on day 20 in *DLK1*-activated samples compared to non-activated controls. **(A)** Relative *DLK1* expression on days 3, 6, and 10 with CRISPR activation. The fold changes are calculated compared to their non-activated counterparts at each time point. When *DLK1* was activated during dSMADi, its expression gradually diminished in time, with overexpression occurring during the first days of activation. **(B)** *DLK1* activation during the dSMADi phase (acc and aaa conditions) resulted in suppression of endogenous *DLK1* expression. In contrast, activation during the FGF8 phase (cac condition) did not suppress *DLK1* expression; instead, elevated DLK1 levels were maintained throughout Dox and TMP treatment. Samples were collected from three independent experiments (*n* = 3). Statistical significance indicated as (*p <.05, **p <.01, **** *p* <.0001).

Supplementary Figure 2 Sample collection schematic for RNA sequencing. Samples were collected on days 10, 20 and 25 after activation during dSMADi, or activation during FGF8 treatment, or activation throughout the differentiation together with their non-activated counterparts.

Supplementary Figure 3 Principal Component Analysis (PCA) of day 25 RNA sequencing samples. PCA plot showing the distribution of four experimental conditions based on gene expression profiles. PC1 (51.99%) explains the largest variance, separating ccc/cac (no activation/*DLK*1 activation during FGF8 phase) from acc/aaa (*DLK1* activation during dSMADi/whole protocol). PC2 (11.60%) represents additional variation. Each point represents a biological replicate, color-coded by condition: Day25_ccc (purple, no activation), Day25_cac (cyan, activation during the FGF8 phase), Day25_acc (green, activation during dSMADi), and Day25_aaa (red, activation throughout differentiation). The clustering pattern indicates that *DLK1* activation timing influences transcriptomic differences, with ccc/cac and acc/aaa grouping together, consistent with our previous observations.

Supplementary Figure 4 qPCR validation of differentially expressed genes. **(A)** *SOX2*,* SPRY2*,* FOXG1*,* LHX2*,* DMRTA1*,* DIO3*,* FGF8*,* FZD5*, and *RAX* were downregulated in day 10 *DLK1* activated cells compared to non-activated condition. **(B)** *DLX5*,* SLC32A1*,* and GAD1* were downregulated in 10-day activated D20 cells compared to the non-activated condition. In contrast, *RSPO2*,* WNT5A* and *LMX1A* were upregulated with activation. Samples were collected from three independent experiments (*n* = 3). Statistical significance indicated as (*p <.05, **p <.01, ***p <.001).

Supplementary Figure 5 Original annotated western blot images generated with anti-DLK1 antibodies (Santa Cruz sc-376755 and Abcam ab21682). The areas inside the red boxes are the relevant lanes.

Supplementary Table 1 List of the qPCR primer used in this study.

Supplementary Table 2 List of the antibodies used in this study.

Supplementary Table 3 List of DEGs on day 10 (acc vs ccc).

Supplementary Table 4 List of DEGs on day 20 (acc vs ccc).

Supplementary Table 5 List of DEGs on day 25 (acc vs ccc).

## Data Availability

Detailed data is available upon request.
