# Supplementary figures and images for: Dual Role of DLK1 in GnRH Neuron Ontogeny
Source: Stem Cell Rev Rep. 2025 Sep 9;21(8):2711–26. doi: 10.1007/s12015-025-10972-y (PMC12504412; doi:10.1007/s12015-025-10972-y)

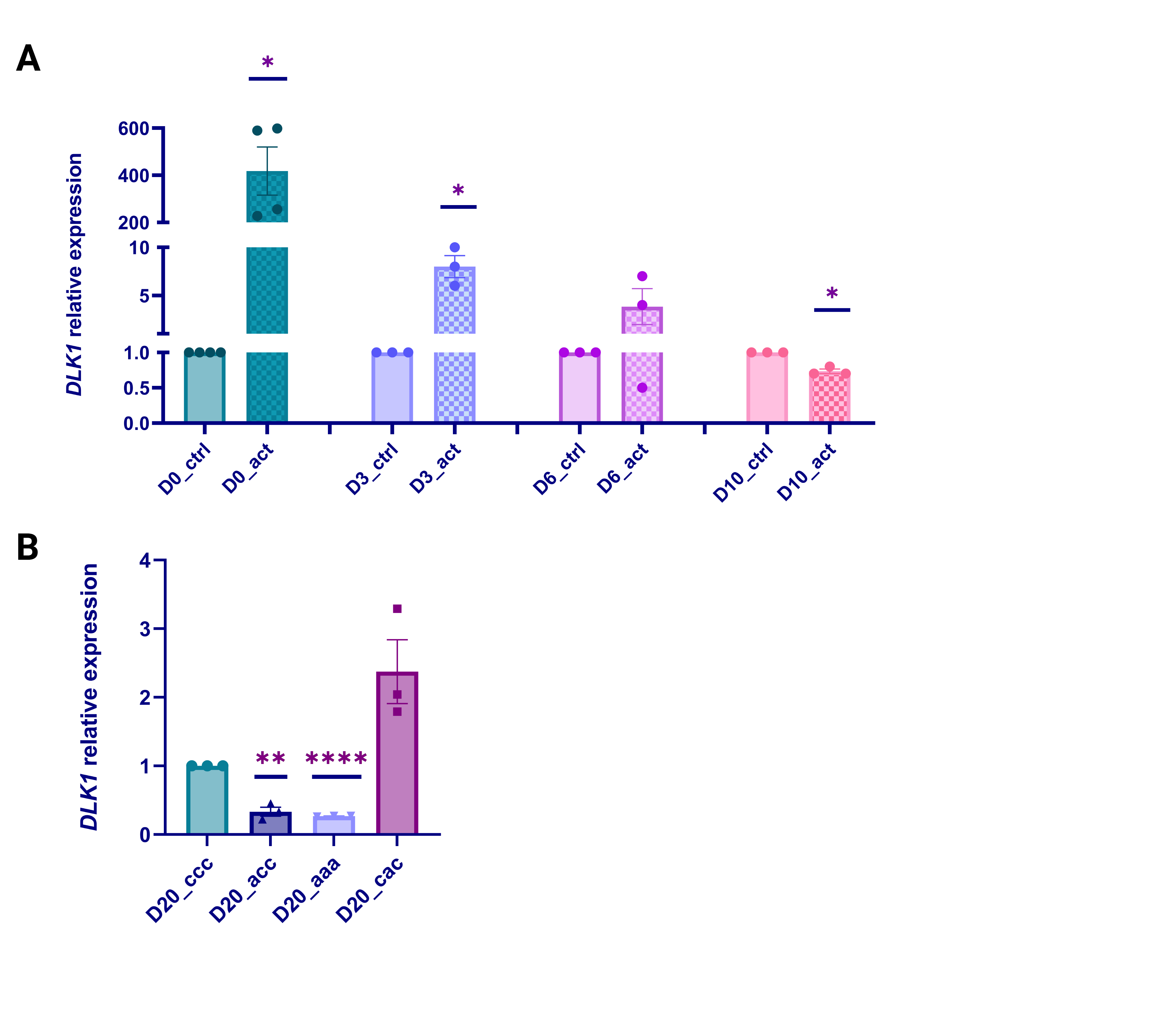

Supplement: Supplementary file 1 — DLK1 expression during dSMADi and on day 20 in DLK1-activated samples compared to non-activated controls. (A) Relative DLK1 expression on days 3, 6, and 10 with CRISPR activation. The fold changes are calculated compared to their non-activated counterparts at each time point. When DLK1 was activated during dSMADi, its expression gradually diminished in time, with overexpression occurring during the first days of activation. (B) DLK1 activation during the dSMADi phase (acc and aaa conditions) resulted in suppression of endogenous DLK1 expression. In contrast, activation during the FGF8 phase (cac condition) did not suppress DLK1 expression; instead, elevated DLK1 levels were maintained throughout Dox and TMP treatment. Samples were collected from three independent experiments (n = 3). Statistical significance indicated as (*p <.05, **p <.01, **** p <.0001). [file 12015_2025_10972_MOESM1_ESM.png]

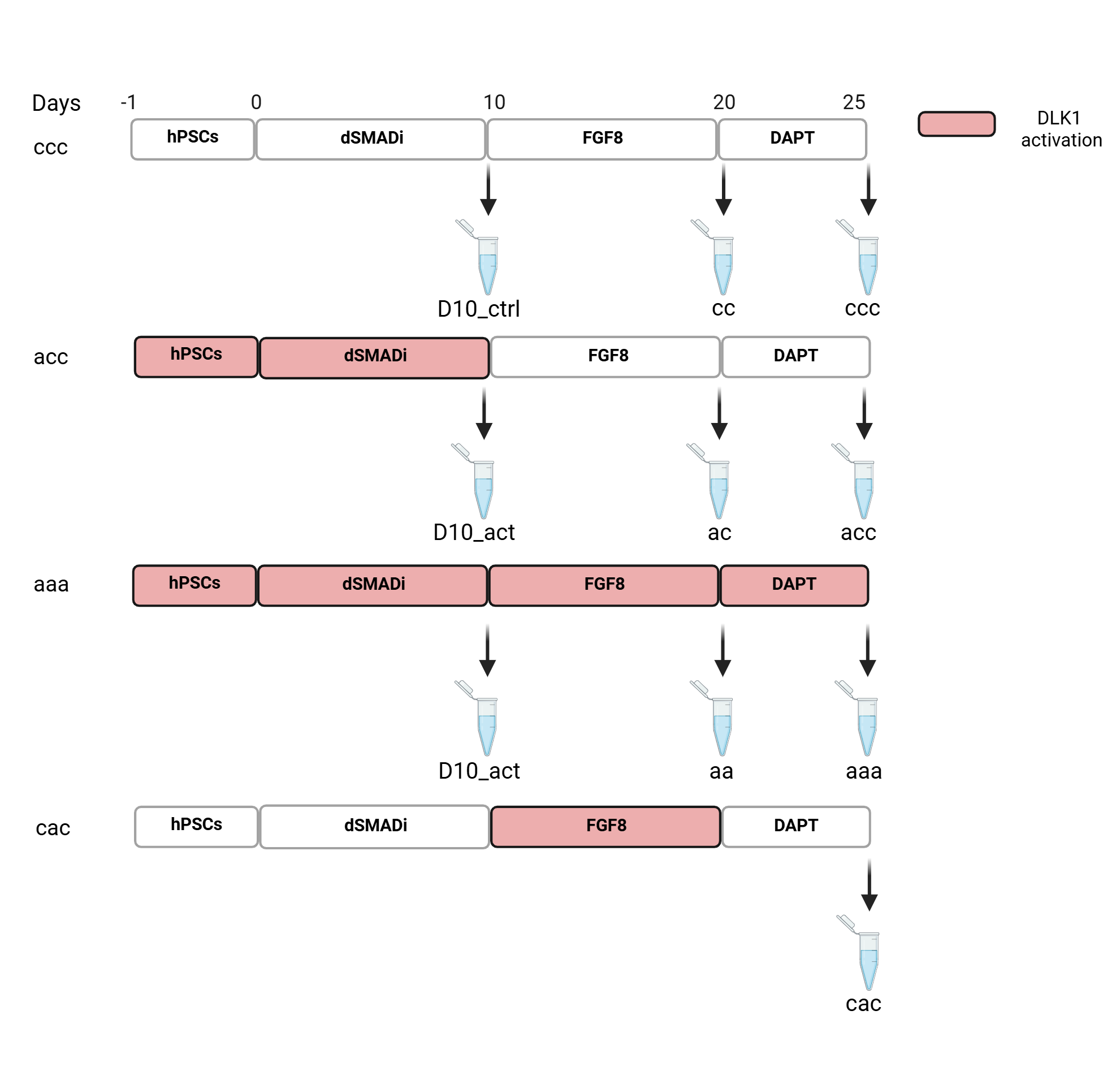

Supplement: Supplementary file 2 — Sample collection schematic for RNA sequencing. Samples were collected on days 10, 20 and 25 after activation during dSMADi, or activation during FGF8 treatment, or activation throughout the differentiation together with their non-activated counterparts. [file 12015_2025_10972_MOESM2_ESM.png]

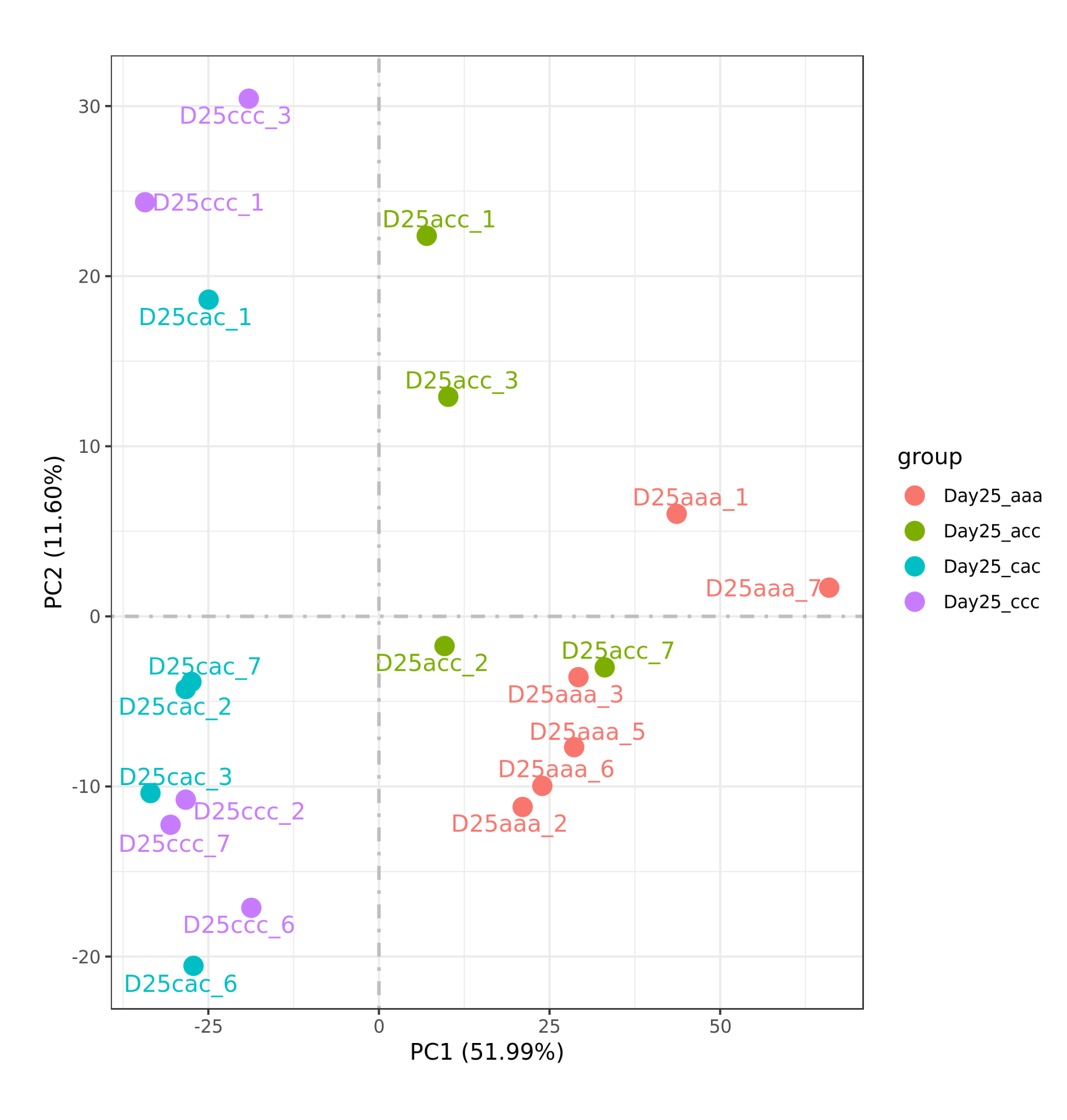

Supplement: Supplementary file 3 — Principal Component Analysis (PCA) of day 25 RNA sequencing samples. PCA plot showing the distribution of four experimental conditions based on gene expression profiles. PC1 (51.99%) explains the largest variance, separating ccc/cac (no activation/DLK1 activation during FGF8 phase) from acc/aaa (DLK1 activation during dSMADi/whole protocol). PC2 (11.60%) represents additional variation. Each point represents a biological replicate, color-coded by condition: Day25_ccc (purple, no activation), Day25_cac (cyan, activation during the FGF8 phase), Day25_acc (green, activation during dSMADi), and Day25_aaa (red, activation throughout differentiation). The clustering pattern indicates that DLK1 activation timing influences transcriptomic differences, with ccc/cac and acc/aaa grouping together, consistent with our previous observations. [file 12015_2025_10972_MOESM3_ESM.png]

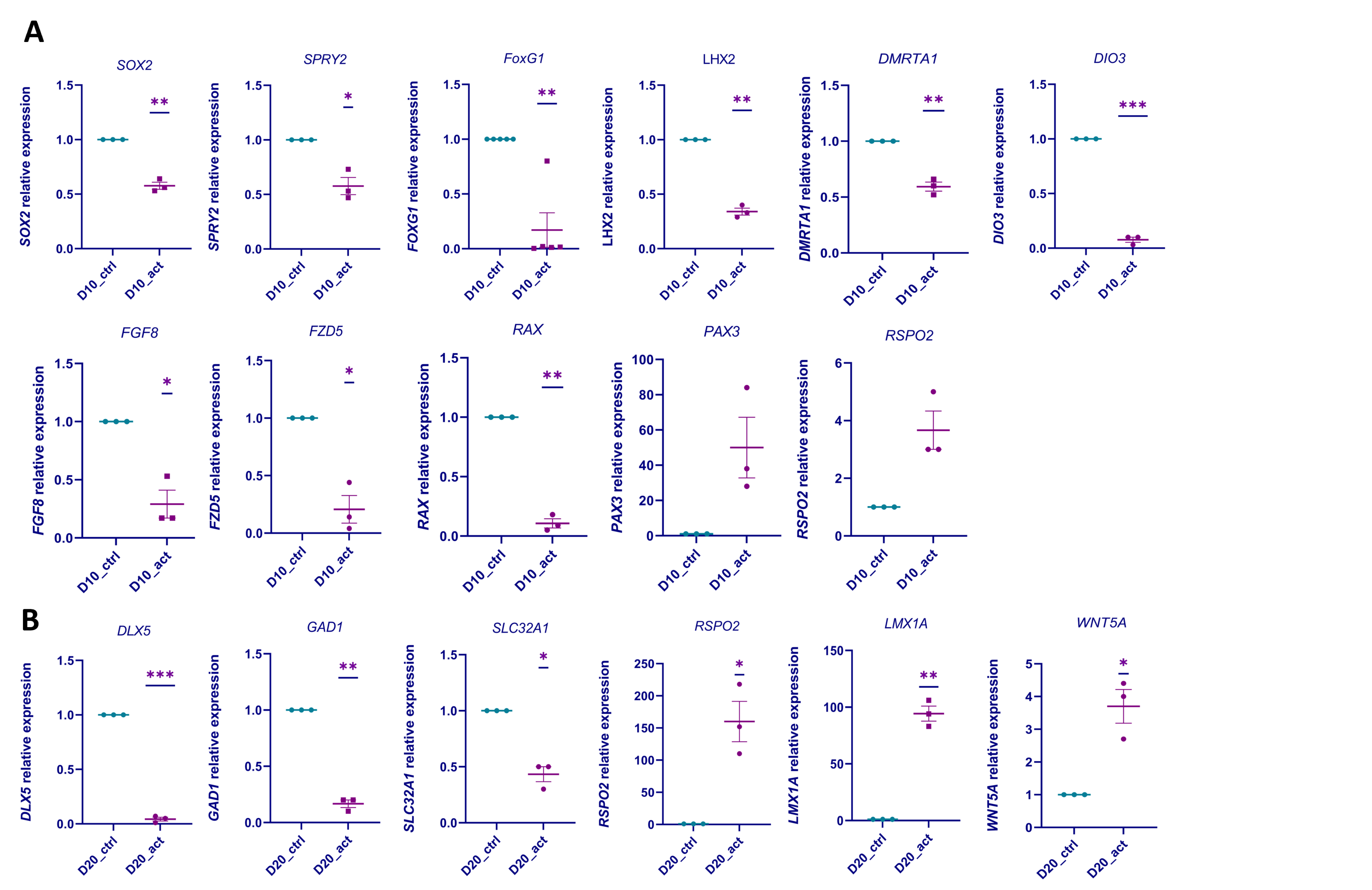

Supplement: Supplementary file 4 — qPCR validation of differentially expressed genes. (A) SOX2, SPRY2, FOXG1, LHX2, DMRTA1, DIO3, FGF8, FZD5, and RAX were downregulated in day 10 DLK1 activated cells compared to non-activated condition. (B) DLX5, SLC32A1, and GAD1 were downregulated in 10-day activated D20 cells compared to the non-activated condition. In contrast, RSPO2, WNT5A and LMX1A were upregulated with activation. Samples were collected from three independent experiments (n = 3). Statistical significance indicated as (*p <.05, **p <.01, ***p <.001). [file 12015_2025_10972_MOESM4_ESM.png]

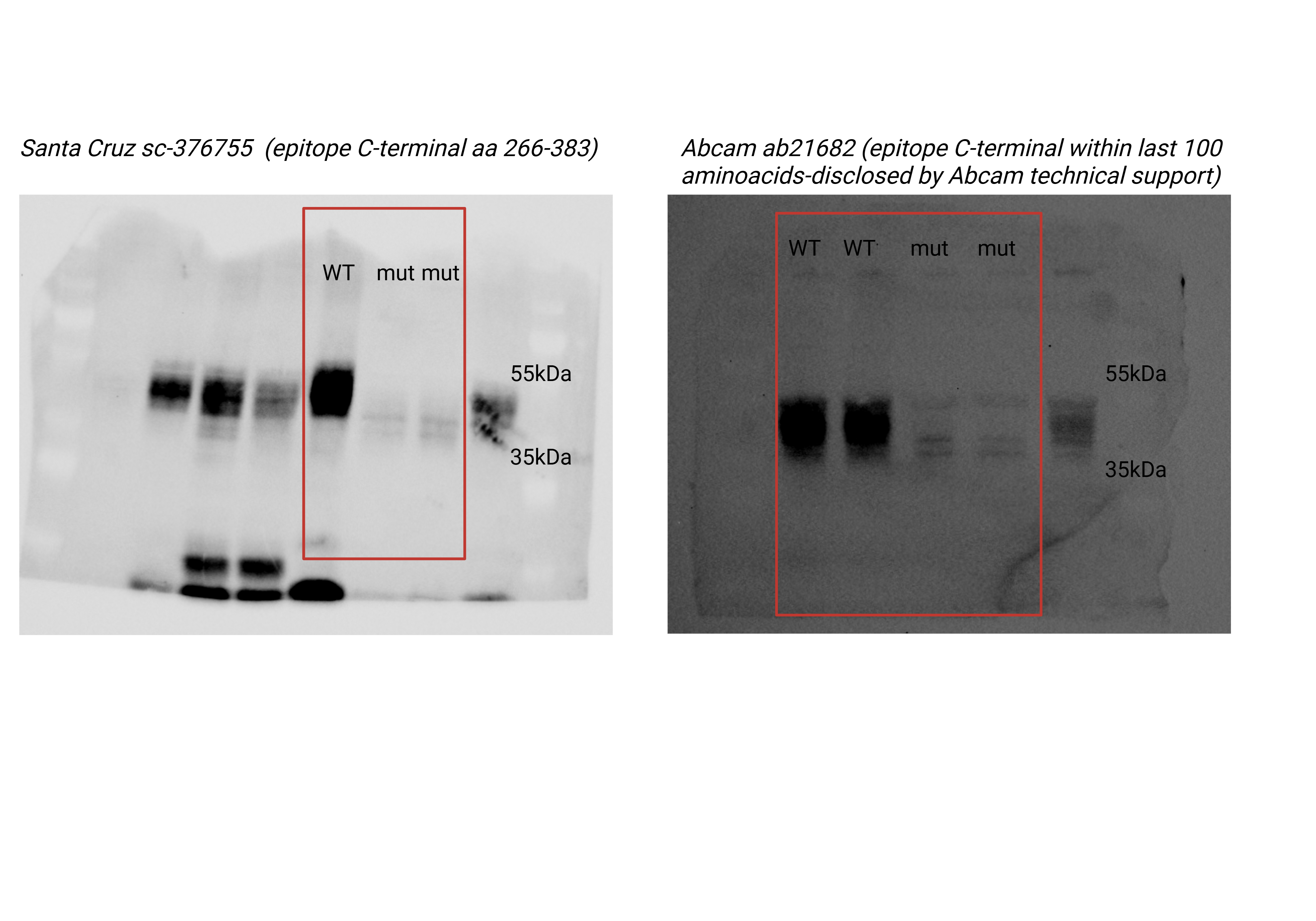

Supplement: Supplementary file 5 — Original annotated western blot images generated with anti-DLK1 antibodies (Santa Cruz sc-376755 and Abcam ab21682). The areas inside the red boxes are the relevant lanes. [file 12015_2025_10972_MOESM5_ESM.png]

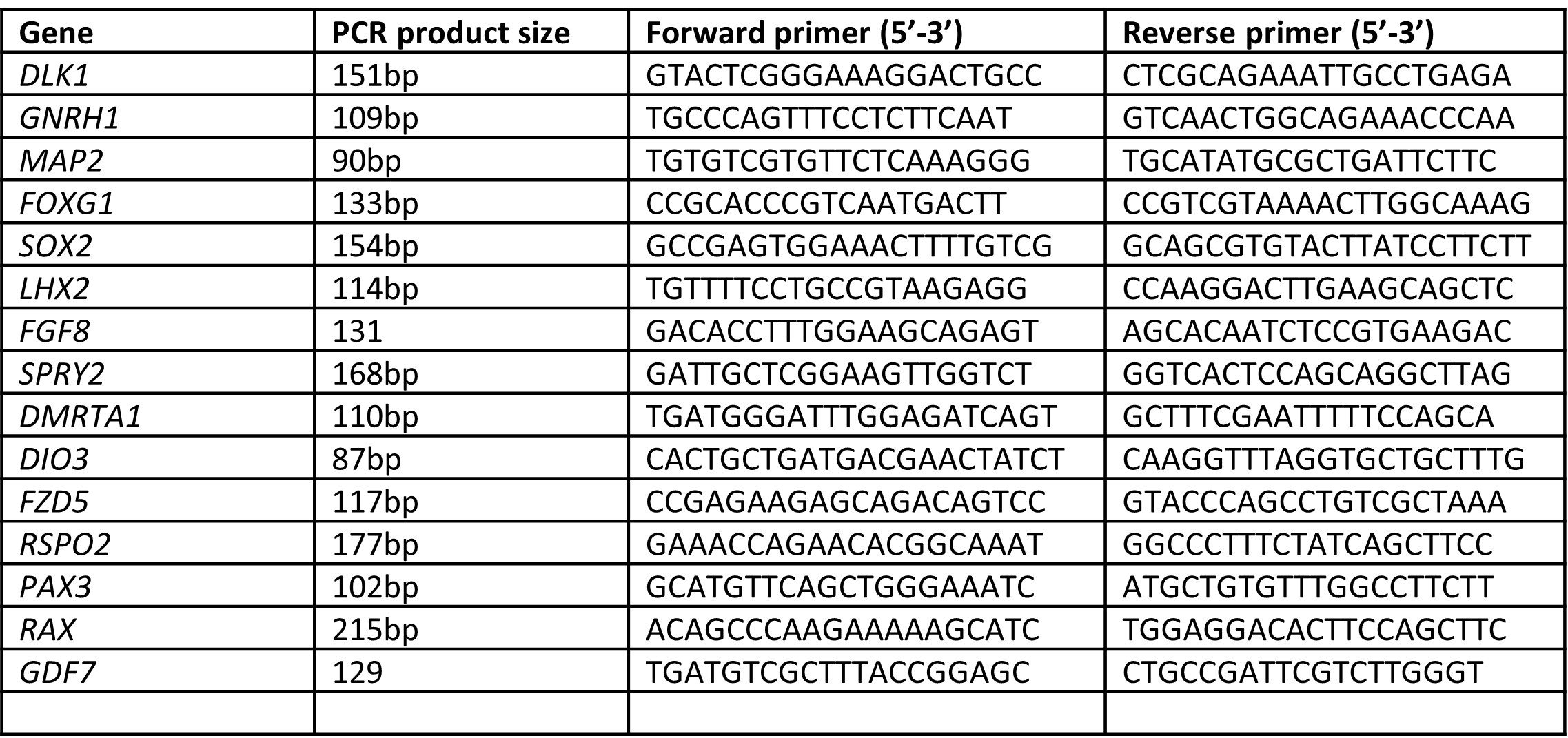

Supplement: Supplementary file 6 — List of the qPCR primer used in this study. [file 12015_2025_10972_MOESM6_ESM.png]

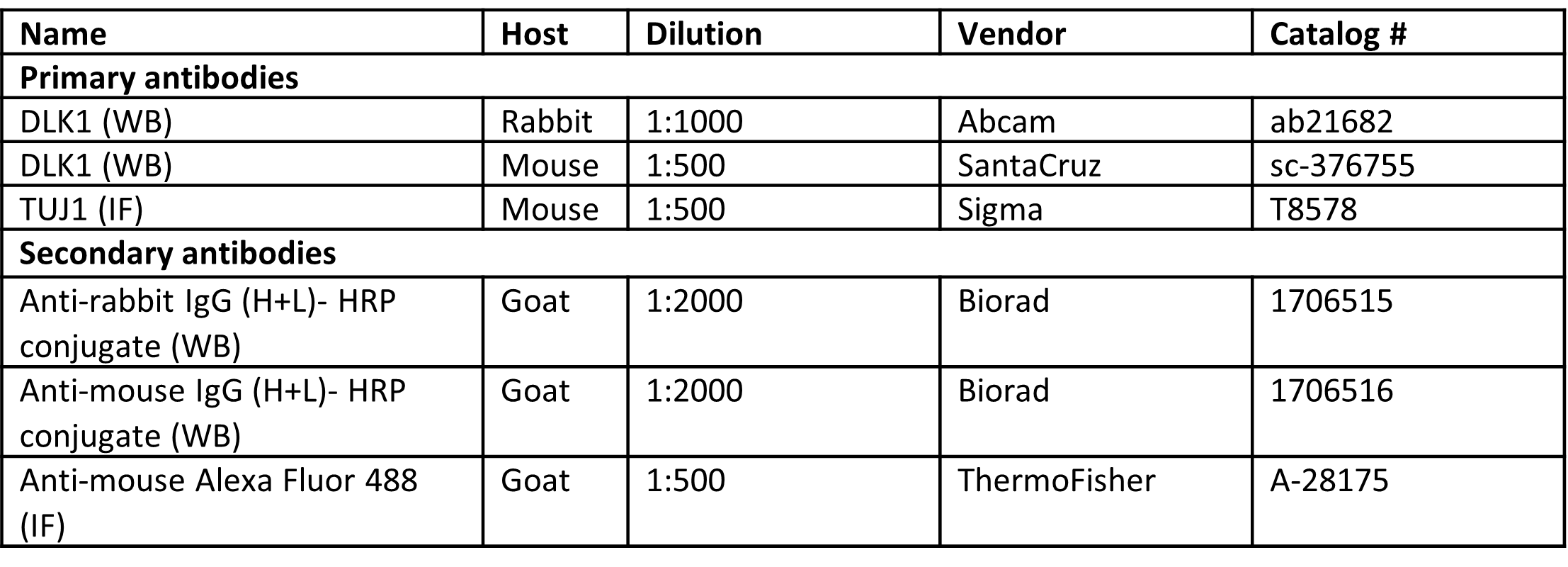

Supplement: Supplementary file 7 — List of the antibodies used in this study. [file 12015_2025_10972_MOESM7_ESM.png]
